# Bronchial Mucoepidermoid Carcinoma in a Pediatric Patient: A Case Report

**DOI:** 10.7759/cureus.91701

**Published:** 2025-09-06

**Authors:** Nhan Vo, Van Phu Tran, Gia Bao Phung, Nghia P Nguyen

**Affiliations:** 1 Respiratory Medicine Department, Ho Chi Minh City Children Hospital, Ho Chi Minh City, VNM; 2 International Medical Faculty, Nam Can Tho University, Can Tho City, VNM

**Keywords:** bronchial tumor, case report, histopathology, mucoepidermoid carcinoma, pediatric lung cancer, surgical management

## Abstract

Pulmonary mucoepidermoid carcinoma (MEC) is relatively common in adults but rare in children. Originating from the submucosal glands of the tracheobronchial tree, these tumors manifest with cough, hemoptysis, recurring fever, and pneumonia. Herein, we report a case of a nine-year-old girl who presented to our department with a persistent cough and pneumonia. Upon failure of the initial empirical treatment, computed tomography (CT) showed an endobronchial mass originating from the left main bronchus. Bronchoscopy confirmed the presence of a multifocal, inconsistent mass with smooth borders blocking the left main bronchus near the carina. Histopathological biopsy revealed a low-grade MEC. The patient underwent resection of the left main bronchus with end-to-end anastomosis. Postoperative treatment for pneumonia was provided, and she was discharged from the hospital with recommendations for adjuvant chemotherapy. However, the patient was lost to follow-up. To our knowledge, this is the first documented pediatric bronchial MEC in the Southeast Asian population. It highlights the diagnostic challenges and underscores the importance of early recognition and intervention.

## Introduction

Primary lung cancer in children is a rare occurrence and exhibits a distinct differential from that observed in adults. Among bronchial tumors, the most common histological subtypes are mucoepidermoid carcinoma (MEC), carcinoid tumor, and adenoid cystic carcinoma [1,2]. The etiology of these malignancies remains unknown [1]. Pulmonary salivary gland-type tumors, including MEC, account for approximately 0.1-0.2% of all lung cancers and 1-5% of bronchial adenomas [1,2]. The location of the tumor can result in a range of symptoms, including cough, hemoptysis, recurring fever, and pneumonia [2]. However, up to one-third of individuals may not experience any symptoms at all [1,2]. With a predilection for lobar bronchi over the trachea or mainstem bronchi, MEC frequently develops in relation to the tracheobronchial tree [1].

To the best of our knowledge, there have been no reported cases of MEC among Southeast Asian children. Herein, we present a case of pediatric bronchial MEC diagnosed in a nine-year-old patient from Vietnam. Furthermore, we discuss the potential risk factors and implications for early detection and intervention, acknowledging the importance of timely management in improving patient outcomes.

## Case presentation

**Figure 1:**
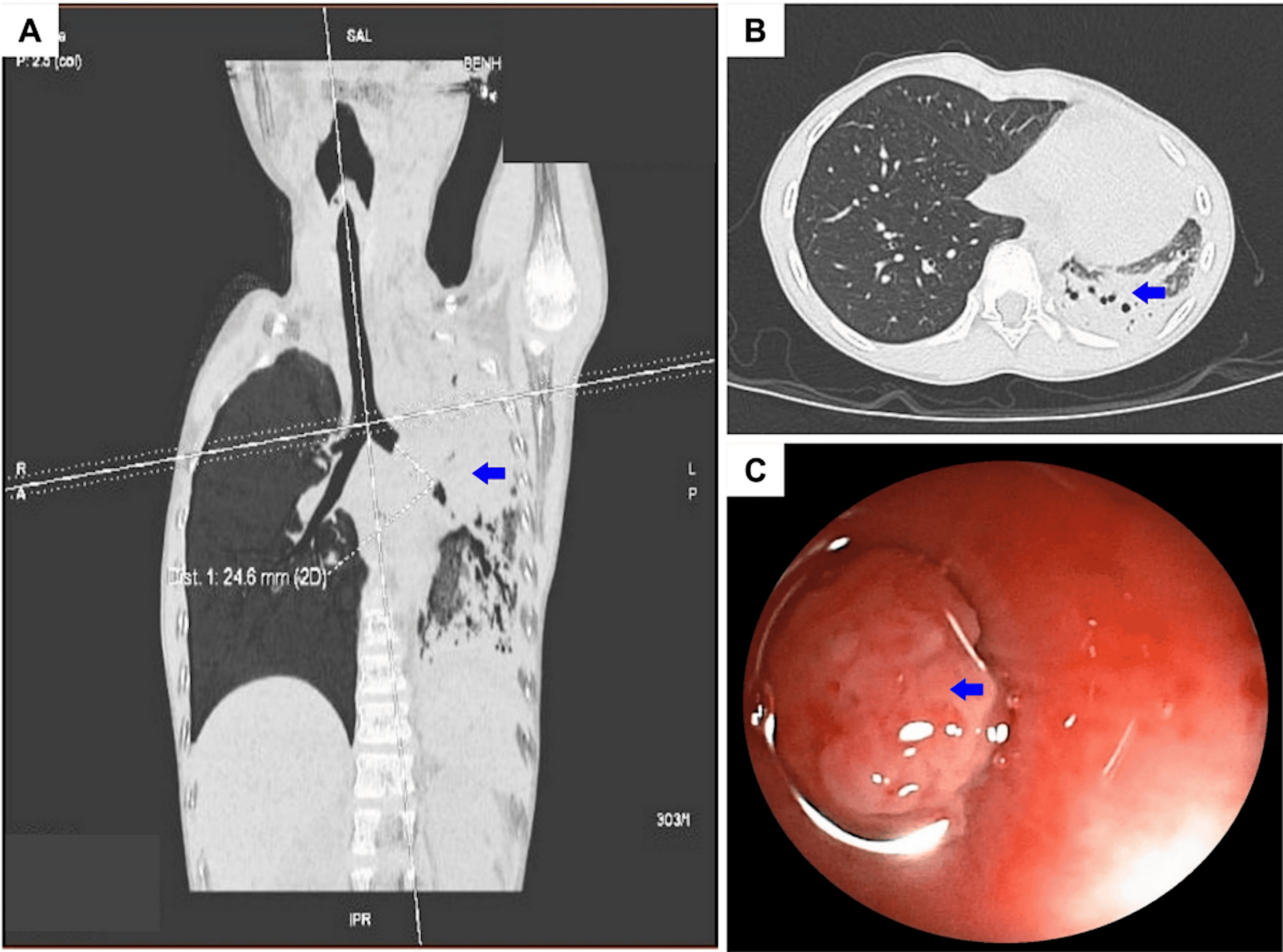
Computed tomography (CT) scan of the patient. (A) Endobronchial mass originating from the left main bronchus, causing significant compression of the left main bronchus and resulting in almost complete collapse of the left lung; (B) Left lung showing enlarged mediastinal lymph nodes, consolidation in the lower lobe, bronchiectasis, and evidence of air trapping; (C) Multifocal mass blocking the lumen of the left main bronchus as seen in bronchoscopy.

A nine-year-old girl presented to our department with a persistent productive cough for a week and complained of shortness of breath and fever. No abnormalities were identified in the patient's blood work or exposure history. Due to the concurrence of these specific symptoms, the patient was initially diagnosed with community-acquired pneumonia and was treated accordingly. However, despite standard empirical antibiotic therapy for community-acquired pneumonia, the patient did not experience any improvement. A decision was taken to conduct a more comprehensive evaluation using computed tomography (CT). The CT scan revealed a 24.6 mm enhancing endobronchial mass originating from the left main bronchus, causing significant compression of the left main bronchus and resulting in partial collapse of the left lung (Figure 1).

**Figure 2:**
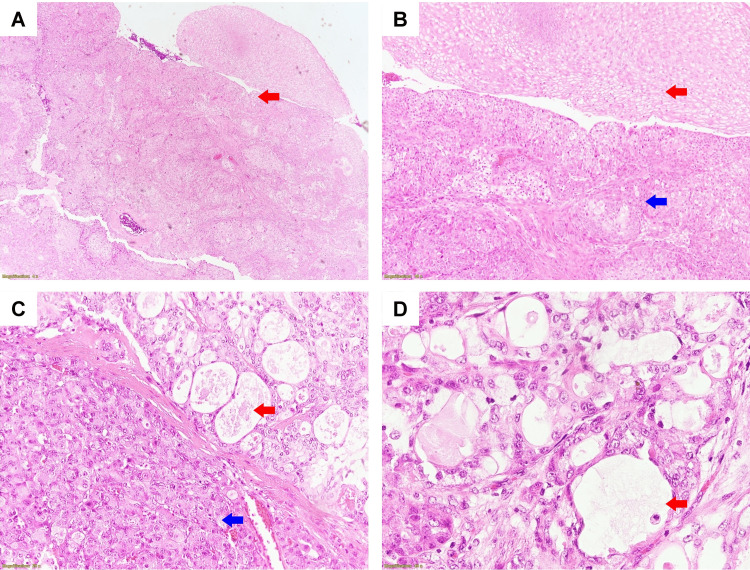
Light microscopy examination of the biopsy sample, showing a low-grade mucoepidermoid carcinoma, using hematoxylin and eosin (H&E) staining. (A) Low-grade mucoepidermoid carcinoma (red arrow); H&E, original magnification 40×; (B) Epidermal structure visualized in the upper half (red arrow) and intermediate structure visualized in the lower half (blue arrow); H&E, original magnification, 100×; (C) Mucoid structure seen in the upper right part (red arrow) and intermediate structure seen in the lower left part (blue arrow); H&E, original magnification, 200×; (D) Mucus-like structures are visualized (red arrow); H&E, original magnification 400×.

Additionally, the CT showed the enlargement of mediastinal lymph nodes, consolidation in the left lower lobe, bronchiectasis, and evidence of air trapping. Considering the characteristics observed in the imaging, the primary differential diagnoses under consideration included a carcinoid tumor, as well as other rarer endobronchial conditions. The possibility of a chronic foreign body obstruction was not definitively ruled out at this point. Bronchoscopy verified the presence of a multifocal, inconsistent mass with smooth borders within the lumen of the left main bronchus, resulting in the complete blockage of the left main bronchus near the carina (Figure 1). A biopsy sample was sent for histopathological evaluation during the bronchoscopy. Subsequent histopathological analysis showed the tissue demonstrating distinctive features of a low-grade bronchial MEC (Figure 2).

**Figure 3:**
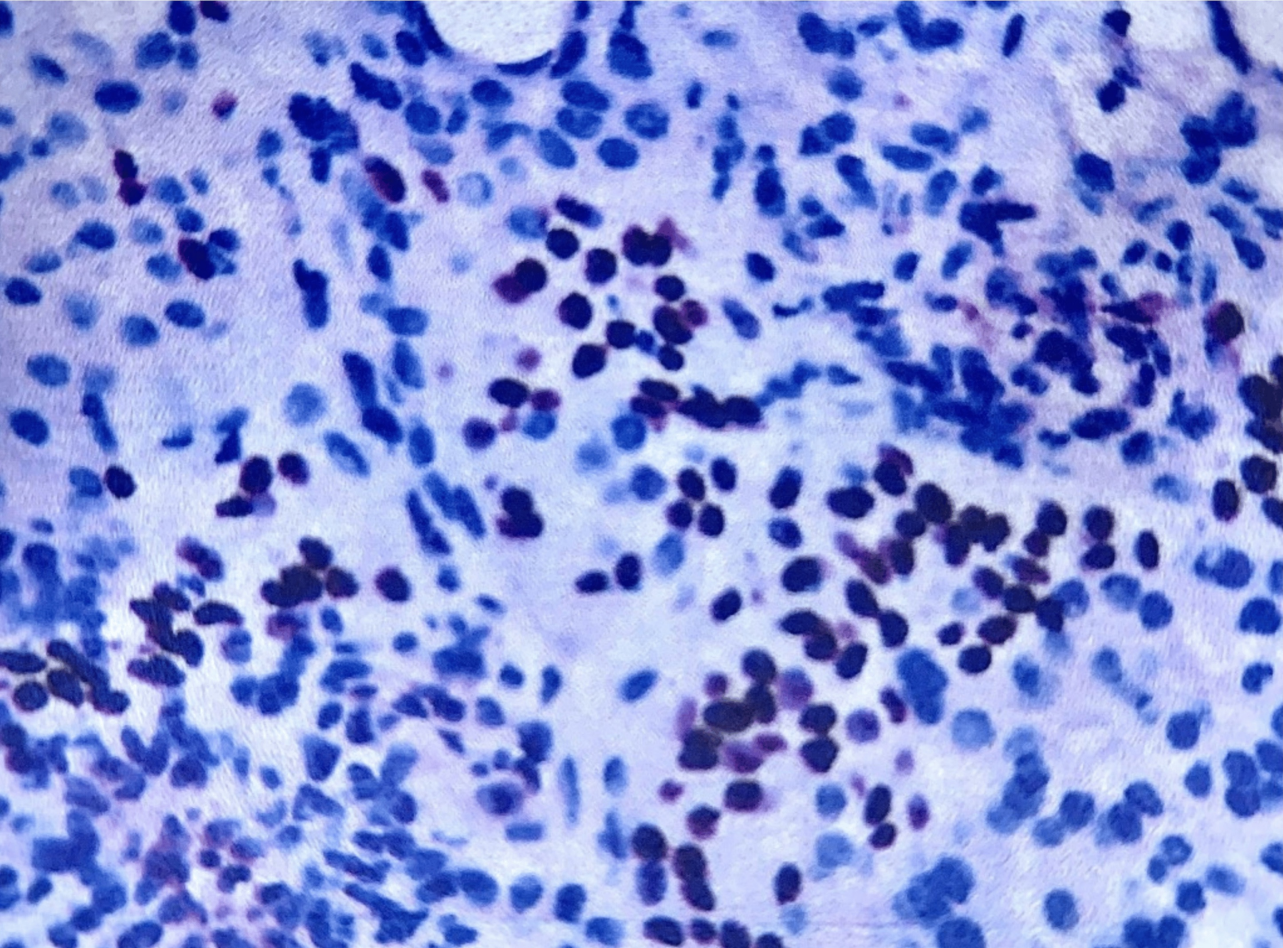
Low-grade mucoepidermoid carcinoma visualized using immunohistochemical marker anti-p63 antibody (+). Nuclei of cells of the epidermal structural component are also visualized; original magnification, 400×.

The sample from the tumor site was mainly composed of mucoid components, including large mucus-secreting cells with blue-gray mucus-filled cytoplasm and surrounding lumen-filled cavities containing mucus. Tumor tissue also presented with areas of epidermal structure consisting of clusters of non-keratinized squamous cells with bright cytoplasm, containing glycogen, intercellular bridges but not forming keratin bridges, and a few solid areas consisting of cells with an intermediate level of differentiation between the above two cell types. These intermediate cells were small, with scant cytoplasm, and appeared as bright cells. Tumor cells had small, uniform nuclei and little cell division. No areas of necrosis or bleeding were recorded on the surveyed tumor sample. The Periodic Acid-Schiff (PAS) stain was positive with mucus. Immunohistochemical staining with anti-p63 antibody was positive (Figure 3).

The patient subsequently underwent surgical resection of the left main bronchus with end-to-end anastomosis at the oncology department of our hospital and showed no radiological evidence of metastasis. Postoperative management focused on treating pneumonia with intravenous antibiotics. She was discharged from the hospital with a recommendation for follow-up. Chemotherapy was recommended, but the patient did not return for follow-up. Repeated attempts to contact the patient remained unsuccessful.

## Discussion

Historically, the label "bronchial adenoma" referred to a collection of slow-growing tumors arising from bronchial glands, considered less aggressive than bronchogenic carcinomas [3]. MEC, along with adenoid cystic carcinomas, mixed tumors, and carcinoid tumors, was grouped together as bronchial adenoma [1,2]. It is uncommon for mucous and serous glands of the airways to generate tumors that look identical under the microscope to salivary gland cancers of the head and neck [4].

However, primary salivary-type tumors of the lung (PSGTTL) were later recognized as a primary group of neoplasms. On histology, these tumors closely resemble salivary gland cancers, and they are thought to develop from submucosal glands within the tracheobronchial tree [5]. It is well established that salivary-type neoplasms can occur at multiple organ sites due to the presence of similar structural homologies within these organs [5]. PSGTTL are extremely uncommon, representing under 1% of all primary pulmonary malignancies [2]. A study conducted by Kang et al. revealed that among the PSGTTL, MEC, adenoid cystic carcinoma, and epithelial-myoepithelial carcinoma are the three most common histological subtypes [4].

MEC is more frequently encountered in the salivary glands, particularly the parotid and submandibular glands. Additionally, it has been reported in minor salivary glands of the oral cavity and perimaxillary region. Pulmonary MEC is rare, with an estimated incidence of only 0.1%-0.2% of lung cancers [6]. MEC occurs in patients with an age range of 3 to 78 years. Several cases have been reported among children, accounting for 10% of this age group [7]. MEC typically presents as an intraluminal mass obstructing the airways, most commonly the segmental and lobar bronchi [7,8]. Airway obstruction consequently leads to complete or partial atelectasis of the corresponding distal parenchyma, with subsequent obstructive irritation and inflammation [8]. This leads to patients complaining about cough, hemoptysis, wheezing, bronchitis, chest pain, and fever. From a clinical perspective, this case underscores the importance of considering endobronchial tumors such as MEC in children who present with recurrent or refractory pneumonia. Persistent symptoms despite standard empirical therapy should prompt further investigation with CT imaging and bronchoscopy, since early suspicion and timely diagnostic workup are critical to avoiding delayed diagnosis.

Given the slow tumor growth and development, signs and symptoms often persist for months or years with no complete resolution with symptomatic treatment. Recurrent pneumonia that does not resolve with standard therapy should always prompt further evaluation for possible endobronchial lesions such as MEC. Chest X-rays and CT scans are the main imaging tools for MEC. CT images are essential for identifying the characteristics of tumors, such as their margins, shape, density, and pattern of enhancement [9]. Radiologically, MEC often presents as a rounded or ovoid lesion with clearly demarcated, smooth edges [9,10]. Radiological findings often include features of bronchial narrowing or blockage, such as distal airway dilatation, mucus plugging, post-obstructive pneumonia, air trapping, collapse of lung segments, or peripheral hyperlucency [8].

Ishizumi et al. reported that MEC lesions often exhibit strong contrast enhancement on high-resolution computed tomography (HRCT), with attenuation values significantly exceeding those of the chest wall muscles [9]. In contrast, Kim et al. described MEC tumors with only mild contrast uptake on HRCT scans [10]. The diverse characteristics of MEC on imaging can be caused by the presence of abundant micro-tumor vessels [9]. The differential diagnosis of MEC includes lung malignancies such as squamous cell carcinoma, adenocarcinoma, or carcinoid tumor. However, differentiating tumors can be challenging clinically and radiologically due to the varied heterogeneity in their characteristics. Nevertheless, an ovoid mass with smooth margins on imaging should raise suspicion for a MEC.

Flexible bronchoscopy allows direct visualization of intraluminal tumors and remains the main diagnostic tool for MEC. Peripheral and extraluminal tumors, however, are not visualized by bronchoscopy. Bronchoscopically, MEC often presents as a soft, exophytic polypoid lesion with a broad base, resembling the surrounding bronchial mucosa in color. It may also be found as pedunculated with a well-formed stalk [6,9,11]. The cut surface is gray-white-tan colored with a glistening mucoid texture. Cystic degeneration may be appreciated in some MECs [6]. It should be noted that due to the rich vascularity of the tumor, bronchoscopy and biopsy carry a significant risk of hemorrhage. Rigid bronchoscopy can be used to avoid hemorrhage and further complications [8].

Microscopically, MECs are graded as either low or high grade, using criteria adapted from salivary gland MEC classification [12]. Low-grade tumors usually behave in a relatively indolent manner and have an excellent prognosis after resection, whereas high-grade tumors are associated with poorer outcomes and higher mortality. These tumors typically consist of three principal cell types: mucus-secreting, intermediate, and squamous cells, with the frequency and characteristics of the components serving as the main tool to grade these tumors [2,6,12]. Immunophenotyping may aid in the differential diagnosis of MEC. Cytokeratin 7 (CK-7) and thyroid transcription factor (TTF-1) have been described as two useful markers. TTF-1 can be used to differentiate MEC from adeno-squamous carcinoma. TTF-1 and surfactant are commonly positive in adeno-squamous carcinoma, while they are always negative in high-grade MEC of the lung [6,13]. On the other hand, CK-7 is found to be positive in MEC tumor cells [6,7].

The prognosis of MEC depends on various factors, mostly age, tumor grade, lymph node metastasis, and tumor-node metastasis staging. An analysis of 21 patients with primary lung MEC revealed that patients aged 60 years or older have poorer overall survival [13]. In addition, metastasis to regional lymph nodes and atypical mitotic activity are more commonly seen among high-grade tumors. Low-grade tumors mainly consist of cystic areas and are less invasive, though extension to the bronchial mucosa and pulmonary parenchyma has been observed in some cases. Additionally, an unusual reaction of dense eosinophilic sclerosis resembling amyloid that curved around nests of infiltrating tumors may also be seen among low-grade tumors [12].

Surgical removal serves as the treatment of choice for most MEC cases, especially those of low grade, with no distant metastasis or an unresectable lesion [7,8,12,14]. Surgical options include pneumonectomy, lobectomy, bi-lobectomy, and sleeve resection, accompanied by lymph node dissection. Sleeve resection and pneumonectomy are usually reserved for central and more aggressive lesions. Besides, a few cases of MEC have been treated with bronchotomy or bronchoplasty. Open or video-assisted surgical removal is a common option, as endoscopic removal carries a risk of hemorrhage and/or incomplete tumor resection. Radiation therapy or chemotherapy alone, preoperatively or postoperatively, has been reserved for more aggressive and unresectable cases of MEC. Nonetheless, the effectiveness of radiation therapy and chemotherapy in pulmonary MECs remains unclear and requires further study [8].

The key strength of our report is that it represents, to our knowledge, the first documented pediatric bronchial MEC in the Southeast Asia region. This adds valuable information to the global literature and highlights the need for greater awareness among clinicians in similar settings. A limitation of our case is the loss to follow-up and the absence of a postoperative chest X-ray, which prevented assessment of long-term outcomes and limited radiological documentation of recovery. These challenges highlight the importance of systematic follow-up in rare pediatric malignancies.

## Conclusions

MEC remains a particularly challenging diagnosis in the pediatric population because of its rarity and its non-specific clinical manifestations. Imaging modalities, such as CT, are invaluable in identifying obstructive endobronchial masses, while bronchoscopy enables direct visualization and tissue sampling, albeit with procedural risks due to the tumor's vascularity. Surgical excision remains the cornerstone of treatment for low-grade MEC, offering favorable outcomes when performed promptly and completely.

Our case highlights the need to maintain a high index of suspicion for MEC in children with recurrent or refractory pneumonia, particularly when imaging suggests an obstructive endobronchial lesion. With pediatric cases still scarce, particularly in Southeast Asia, systematic reporting and multicenter collaboration are needed to clarify optimal management, long-term outcomes, and the role of adjuvant therapy.
